# The Combined Effect of Birth Weight and Lifestyle on Clustered Cardio-Metabolic Risk Factors in Children and Adolescents: A National School-Based Cross-Sectional Survey

**DOI:** 10.3390/nu14153131

**Published:** 2022-07-29

**Authors:** Di Shi, Jiajia Dang, Ning Ma, Yunfei Liu, Panliang Zhong, Shan Cai, Yinghua Ma, Zhiyong Zou, Yanhui Dong, Yi Song, Jun Ma

**Affiliations:** 1 Institute of Child and Adolescent Health, School of Public Health, Peking University, Beijing 100191, China; 2111210123@bjmu.edu.cn (D.S.); dangjj@bjmu.edu.cn (J.D.); mnmaning@bjmu.edu.cn (N.M.); liuyunfei_pku@163.com (Y.L.); 1610306219@pku.edu.cn (P.Z.); 1710306207@pku.edu.cn (S.C.); yinghuama@bjmu.edu.cn (Y.M.); harveyzou2002@bjmu.edu.cn (Z.Z.); majunt@bjmu.edu.cn (J.M.); 2 National Health Commission Key Laboratory of Reproductive Health, Peking University, Beijing 100191, China

**Keywords:** children, adolescents, cardio-metabolic risk factors, birth weight, lifestyle

## Abstract

Background: Due to the adverse effects of cardio-metabolic risk factors (CMRFs) in children and adolescents on their current and later life health, and the growing evidence that birth weight and lifestyle have on CMRFs, we aimed to estimate the combined effect of birth weight and lifestyle on clustered CMRFs in children and adolescents. Methods: We enrolled 11,509 participants aged 7–18 years old in a national school-based cross-sectional study in seven provinces in China in 2013. Information on CMRFs was collected through anthropometric measurements and blood sample testing. Information on birth weight, lifestyle and other basic information were investigated through children and adolescents’ as well as parents’ questionnaires. The generalized linear mixed model was applied to estimate the odd ratio (OR) and 95% confidence interval (95% CI) for the associations between CMRFs, clustered CMRFs and birth weight, lifestyle, and the combinations of birth weight and lifestyle. Results: Overall, the prevalence of clustered CMRFs was 3.6% in children and adolescents aged 7–18 years, higher in boys (4.4%) than girls (2.9%). The combination of LBW/ideal lifestyle (OR = 2.00, 95% CI: 1.07–3.72) was associated with higher risk of clustered CMRFs, as well as in adolescents aged 13–18 years and in boys. The combination of HBW/poor lifestyle (OR = 1.74, 95% CI: 1.13–2.68) was related to elevated risk of clustered CMRFs, especially in children aged 7–12 years. Conclusions: CMRFs in Chinese children and adolescents is concerning, ideal lifestyle could weaken the association of birth weight with clustered CMRFs, especially in younger age, indicating that programs to prevent abnormal birth weight or poor lifestyle or both among children and adolescents may reduce CMRFs in China.

## 1. Introduction

Cardiovascular diseases have remained a major health burden in adults [1], and most could stem from childhood and adolescence. Specifically, risk factors and behaviors that accelerated the process of cardiovascular diseases begin in childhood and even in the perinatal period [2]. Considering that some children and adolescents have already had identifiable cardio-metabolic risk factors (CMRFs) including hypertension, impaired fasting glucose, dyslipidemia, abdominal obesity at a very early age [3,4,5,6], plus clustered CMRFs, more than three CMRF items, may strongly predict the occurrence of cardiovascular diseases in adulthood [7,8,9]. Identifying it in vulnerable groups and risk factors that could alter CMRFs status might help reduce the prevalence of cardiovascular disease in the future.

There is evidence that low birth weight (LBW) children are more likely to develop cardio-metabolic diseases such as obesity, hypertension, and insulin resistance [10,11,12,13,14,15] while other studies have only found an association between LBW and hypertension [16,17]. Furthermore, studies have shown that high birth weight (HBW) mainly caused by maternal gestational diabetes may result in increased risk of hypertension, abdominal obesity, and insulin resistance during puberty [18,19,20,21] while other studies have not observed such an association [22,23]. The potential impact of HBW on CMRFs in children and adolescents remains insufficient and controversial. In addition, the age and sex differences between birth weight and CMRFs are unclear [24,25,26,27,28]. Therefore, the association between birth weight and clustered CMRFs and how the association varies across age span and different sexes is still unclear.

It is known that poor lifestyle, such as lack of physical activity [29], unhealthy eating habits [8], screen time [30], and sleep deprivation [31], are closely associated with cardio-metabolic risk factors. The high prevalence of abnormal birth weight occurring in China, which was 5.2% of LBW, 7.4% of HBW in 2013 [32], is coupled with the low prevalence of children and adolescents meeting recommended levels of healthy lifestyles with the economic and dietary pattern transitions [33]. Considering that both birth weight and lifestyle have independent effects on CMRFs, and that birth weight is unlikely to affect the lifestyle of children and adolescents [34,35], to what extent the combinations of birth weight and lifestyle act on the occurrence of CMRFs is scarce, especially in China. We hypothesized that birth weight and lifestyle might influence CMRFs, whereby the effects would be cumulative, ideal lifestyle might weaken the association of birth weight with clustered CMRFs, and such an association might vary between age and sex groups. A national school-based healthy lifestyles investigation conducted in seven provinces provided us an opportunity to assess the effects [36]. In this large representative sample of Chinese children and adolescents, we examined overall CMRFs, and aimed to examine the strength of birth weight and lifestyle on clustered CMRFs alone and as combined effects and the age and sex differences in association.

## 2. Materials and Methods

### 2.1. Study Design and Participants

**Figure 1:**
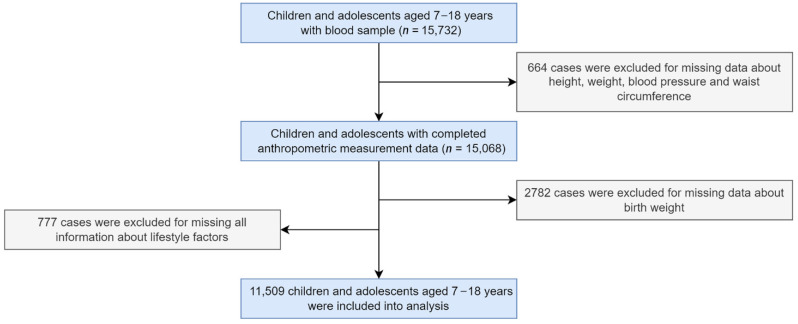
Flow diagram of participants’ inclusion.

The data was extracted from the baseline of a nationwide school-based multi-centered study of 7–18 years children and adolescents which was performed in seven provinces (Tianjin, Shanghai, Chongqing, Liaoning, Hunan, Ningxia, and Guangdong) in China in 2013. The multistage cluster random sampling method was used in this study and more detailed information is published elsewhere [36]. Briefly, three to four districts from each province were randomly selected at first. Second, 12–16 schools were chosen from each district. Third, two to three classes per grade of each school were selected randomly, after excluding children and adolescents with serious organic diseases or those who refused to sign informed consent. Children and adolescents in those classes were invited for blood sample collection. In total, 15,732 participants aged between 7–18 years with blood sample information, while cases with missing data on anthropometric measurement (*n* = 664), missing data on birth weight (*n* = 2782), missing all information about lifestyle factors (*n* = 777) were excluded. A total of 11,509 participants aged 7–18 years were included in the final analysis (Figure 1). The primary sample and the final sample showed no statistical difference in age, residence, and parental education level. However, the proportion of boys in the final sample was less than the primary sample (Table S1). This study has been approved by the Ethical Committee of Peking University (No. IRB0000105213034). All participants and their parents provided signed informed consent.

### 2.2. Study Measurements

#### 2.2.1. Questionnaire Survey

Self-filled questionnaires for children and adolescents were used to obtain basic characteristics of sex, age, residence of children and adolescents, and their lifestyle, such as dietary consumption behaviors (fruits, vegetables, meat, and sugar-sweetened beverages), physical activity level and time, screen time and sleep duration. We collected the frequency and amount of each food consumed in the past week to calculate the dietary behavior of the children and adolescents. Parents self-administered questionnaires were used to obtain birth weight information for children and adolescents. Parents were asked to fill out the questionnaire based on the child’s birth certificate or clinical record. If the documents were not available, they were required recall the information about the child’s early life accurately. Parents were also requested to report the information on single-child status, delivery mode, delivery time, breastfeeding status of the children and adolescents, and parental education level and health behaviors. The questionnaires used in this study were all tested for logic and completeness. An expert panel then reviewed the questionnaires and considered them feasible and acceptable for children and adolescents and their parents.

#### 2.2.2. Anthropometric Measurement and Blood Sample Detection

All children and adolescents received a standardization physical examination [36]. We used a portable stadiometer to measure the height of the participants standing upright without shoes, accurate to 0.1 cm. The participants wore lightweight underwear and weight was measured using a lever scale to the nearest 0.1 kg. Waist circumference was measured 1 cm above the navel, to 0.1 cm. The values of height, weight and waist circumference were the average value of two consecutive measurements.

We used a mercury sphygmomanometer, a stethoscope and an appropriate cuff to measure blood pressure. Children and adolescents were required to quietly rest for 5 min before starting the first measurement. A second measurement was performed after a 5-min interval. We calculated the average systolic and diastolic blood pressure as a result the final measurement of blood pressure. Venous blood was collected by a professional nurse after a 12-h fasting period. Serum glucose, total cholesterol (TC), triglyceride (TG), low-density lipoprotein cholesterol (LDL-C), and high-density lipoprotein cholesterol (HDL-C) were measured using an automatic biochemical analyzer by a qualified biological testing company.

Strict quality control has been carried out in the measurement of this study. All the measuring instruments have been corrected before use, and the measuring personnel have been trained in the standard process. Five percent of the subjects were retested daily, and failure to meet the criteria was considered a failure of the day’s results and required full retesting.

### 2.3. Definition and Classification

#### 2.3.1. Birth Weight

Normal birth weight (NBW) was considered to be children and adolescents with a birth weight from 2500 g to 3999 g. LBW was considered as birth weight less than 2499 g, HBW was considered as birth weight greater than 4000 g [37].

#### 2.3.2. Lifestyle

Dietary consumption, physical activity, screen time and sleep duration were used to construct lifestyle scores. Healthy dietary consumption was defined as daily intake of fruits of ≥3 servings (one serving is about 100 g), vegetables of ≥4 servings (one serving is about 100 g), meat products of 2–3 servings (one serving is about 50 g), and weekly intake of sugar-sweetened beverage of <1 serving (one serving is about 250 mL) [38]. Adequate physical activity was defined as at least one hour of moderate to high intensity physical activity per day. Screen time was defined as less than two hours per day. Healthy sleep duration was defined as sleeping for more than 9 h per day [39]. Participants were grouped in the ideal lifestyle group if they had at least two or more healthy lifestyle factors, or into the poor lifestyle group if they had only one health lifestyle factor or fewer.

#### 2.3.3. Combination of Birth Weight and Lifestyle

According to the combination of birth weight and lifestyle, the participants were divided into six groups: NBW/ideal lifestyle, NBW/poor lifestyle, LBW/ideal lifestyle, LBW/poor lifestyle, HBW/ideal lifestyle, HBW/poor lifestyle.

#### 2.3.4. Clustered CMRFs

Hypertension was defined as blood pressure ≥ 95th percentile of age-, sex-, and height-specific references recommended by American Academy of Pediatrics in 2017 [40]. Impaired fasting glucose was defined as fasting glucose ≥ 5.6 mmol/L [41]. Dyslipidemia was defined according to the National Heart, Lung, and Blood Institute guidelines for cardiovascular health and risk reduction in children and adolescents: elevated TC was defined as ≥5.2 mmol/L, elevated LDL-C was defined as ≥3.4 mmol/L, abnormal HDL-C defined as ≤1.0 mmol/L, and elevated TG defined as ≥1.1 mmol/L for children below 9 years and ≥1.5 mmol/L for those aged 10 years or older [2]. Participants who met any one of the above criteria were divided into dyslipidemia. Abdominal obesity was defined as waist circumference ≥ 90th percentile of age–sex specific references recommended by National Health Commission of the People’s Republic of China in 2018 [42]. Clustered CMRFs was defined as three or more factors being satisfied among all four items of hypertension, impaired fasting glucose, dyslipidemia, and abdominal obesity [43].

#### 2.3.5. Confounding Variables

In this study, children and adolescents were divided into two age groups: 7–12 years and 13–18 years. Sex, residence, delivery mode, single-child status, parental smoking, and parental education were considered as confounding variables. Parental smoking was defined as either of parents smoked in the past week. Parental education was divided into junior high school and below and senior high school and above, with the highest education level of parents (either mother or father) being considered [44]. Missing values of the covariates (<8.6%) were imputed by multiple imputation. Sensitivity analysis results indicated that the direction and value of the findings before and after imputation were essentially unchanged, suggesting that the imputation variables would not affect the main results.

### 2.4. Statistics Analysis

Categorical variables were shown as number (percentage). Chi-square tests and Fisher’s exact test were used to compare the differences of categorical data between sex groups and the differences of CMRFs and clustered CMRFs in different groups. ANOVA and Dunnett-*t* test were used to compare the differences between combinations of birth weight and lifestyles. A generalized linear mixed model was used to estimate the odds ratio (OR) and 95% confidence interval (CI) for association between CMRFs, clustered CMRFs and birth weight, lifestyle, and their combinations, using the school’s name as the random-effect term to control the cluster effect of schools in different groups. Significance level was accepted at two-tailed *p* < 0.05. All data were analyzed by SPSS 26.0 and R 4.1.1.

## 3. Results

### 3.1. The Characteristics of Participants

The basic demographic characteristics of the total 11,509 participants from different sex groups were shown in Table 1. Children and adolescents with LBW accounted for 3.8% and HBW accounted for 9.0%, while poor lifestyle accounted for 46.2%. Girls tended to have unhealthier lifestyle (*p* = 0.005). Among the six combinations, the proportion of the number from high to low was NBW/ideal lifestyle (47.2%), NBW/poor lifestyle (40.1%), HBW/ideal lifestyle (4.7%), HBW/poor lifestyle (4.3%), LBW/ideal lifestyle (2.0%), LBW/poor lifestyle (1.8%). The prevalence of clustered CMRFs in children and adolescents aged 7–18 years was 3.6%, while 4.4% in boys and 2.9% in girls, the sex difference was statistically significant (*p* < 0.001).

### 3.2. CMRFs and Clustered CMRFs in Different Groups

As shown in Table 2, CMRFs and clustered CMRFs were differed in age and sex groups (*p* < 0.05). The proportion of hypertension, dyslipidemia and abdominal obesity differed significantly between urban and rural children and adolescents (*p* < 0.001). Birth weight and the combinations of birth weight and lifestyle were associated with dyslipidemia and abdominal obesity (*p* < 0.05). Lifestyle was associated with hypertension and dyslipidemia (*p* < 0.05). Higher risk of clustered CMRFs was related to meat intake, sugar-sweetened beverages, and sleep duration (*p* < 0.05).

### 3.3. Multivariate Associations between CMRFs, Clustered CMRFs and Its Risk Factors

The associations between CMRFs, clustered CMRFs and birth weight in mixed effect models are shown in Table 3. After adjusting for confounding factors, LBW was related to elevated risk of hypertension in children and adolescents aged 13–18 years (OR = 1.81, 95% CI: 1.19–2.75) and boys (OR = 1.54, 95% CI: 1.06–2.25). HBW was associated with higher risk of abdominal obesity (OR = 1.66, 95% CI: 1.43–1.92) and was associated with clustered CMRFs in children and adolescents aged 7–12 years (OR = 1.70, 95% CI: 1.12–2.58) and girls (OR = 1.83, 95% CI: 1.19–3.08) (Figures S1–S3). Table 4 shows the associations between CMRFs, clustered CMRFs and lifestyle. A higher risk of hypertension was found in the inadequate sleep duration group in total model (OR = 1.18, 95% CI: 1.02–1.36), children aged 7–12 years (OR = 1.25, 95% CI: 1.07–1.45) and girls (OR = 1.28, 95% CI: 1.03–1.59). Abdominal obesity was associated with excessive screen time (OR = 1.17, 95% CI: 1.06–1.30) and inadequate sleep duration (OR = 1.14, 95% CI: 1.01–1.29) in children and adolescents aged 7–18 years. Poor lifestyle was related to abdominal obesity in children aged 7–12 years (OR = 1.15, 95% CI: 1.02–1.29). Unhealthy dietary consumption was associated with a higher risk of clustered CMRFs in boys (OR = 1.40, 95% CI: 1.01–1.94) and lower risk of clustered CMRFs in girls (OR = 0.68, 95% CI: 0.47–0.97) (Figures S4–S6).

As shown in Table 5, a higher risk of hypertension was found in LBW/ideal lifestyle group compared with NBW/ideal lifestyle in adolescents aged 13–18 years (OR = 2.43, 95% CI: 1.15–5.17) and boys (OR = 2.45, 95% CI: 1.25–4.79). Boys with NBW/poor lifestyle had a higher risk of impaired fasting glucose (OR = 1.65, 95% CI: 1.02–2.67). Abdominal obesity was significant higher in HBW/ideal lifestyle (OR = 1.54, 95% CI: 1.26–1.90) and HBW/poor lifestyle (OR = 1.89, 95% CI: 1.53–2.33) groups in children and adolescents aged 7–18 years. The effect value of the NBW/ideal lifestyle and NBW/poor lifestyle groups in abdominal obesity were statistically different from that of the HBW/poor lifestyle group (*p* < 0.05). In the age group of 7–12 years, abdominal obesity in HBW/ideal lifestyle and HBW/poor lifestyle groups was significantly different (*p* < 0.05). In the age group of 13–18 years, dyslipidemia in the LBW/ideal lifestyle and LBW/poor lifestyle groups was significantly different (*p* < 0.05). Higher risk of clustered CMRFs was found in the HBW/poor lifestyle group in total model (OR = 1.74, 95% CI: 1.13–2.68) and children aged 7–12 years (OR = 2.37, 95% CI: 1.39–4.05). It was also found in the LBW/ideal lifestyle group in total model (OR = 2.00, 95% CI: 1.07–3.72), adolescents aged 13–18 years (OR = 3.57, 95% CI: 1.23–10.37) and boys (OR = 2.78, 95% CI: 1.02–7.62) (Figures S7–S9).

## 4. Discussion

To the best of our knowledge, this was the first nationwide study addressing the effects of the combination of birth weight and lifestyle on clustered CMRFs and age and sex differences among children and adolescents in China. Our findings showed that the combined effect of LBW and ideal lifestyle was associated with clustered CMRFs, especially for those adolescents aged 13–18 years and boys. In addition, the significant combined effect of HBW and poor lifestyle on the clustered CMRFs in children and adolescents was found, especially for those children aged 7–12 years.

Previous studies have shown the association between CMRFs and birth weight in children and adolescents [10,11,12,13,14,15,45,46]. However, few studies focused on clustered CMRFs, possibly due to its low prevalence among children and adolescents. However, our study found that HBW had significant effects on clustered CMRFs in children aged 7–12 years and girls. In addition, the disease risk of the HBW group was higher than the corresponding NBW group regardless of lifestyle in children and adolescents aged 7–18 years, suggesting that HBW might be a health risk population that needs to be concentrated on, especially in younger children and girls. Currently it is believed that the cause of HBW is genetic factors [45] and uterine environmental factors. Maternal intermittent hyperglycemia leads to fetal hyperglycemia and fetal release of insulin, insulin-like growth factor and growth hormone which cause an increase in fetal fat deposition and weight gain [47,48,49]. Maternal abnormal lipid levels might also affect the placental nutrition supply, which prompts fetal overgrowth in early life [50]. Studies have shown that 16% of HBW children develop hypoglycemia within 24 h of life due to a placental glucose supply interruption [51,52]. All these factors might affect adipose tissue and pancreatic cell development, leading to increased BMI and impaired glucose metabolism, thus increasing the risk of obesity and metabolic complications in later life [53,54,55]. Furthermore, many studies support the evidence that LBW has an effect on hypertension in children and adolescents [16,17,56], which are consistent with our findings. For this phenomenon, the most reasonable hypothesis based on trial and clinical evidence is “congenital nephron reduction” [57,58], which means impaired intrauterine development can lead to impaired kidney development. A decrease in the number of nephrons could lead to compensatory hypertrophy, associated intraglomerular hypertension could then lead to glomerulosclerosis and hypertension within years [59,60]. In addition, HBW was confirmed to be associated with a higher risk of abdominal obesity in children and adolescents, which was consistent with the results of multiple other studies [61,62]. Early identification of abnormal birth weight children at health risk and the feasible prevention of CMRFs through lifestyle intervention are of great public health significance for the prevention of cardiovascular disease.

This study showed that the combination of LBW/ideal lifestyle was associated with elevated risk of clustered CMRFs. Due to the limitation of LBW sample size, this association was not found in LBW/poor lifestyle. However, it could be seen that the association between LBW and clustered CMRFs was mainly due to its association with hypertension, and studies have shown that the effect of LBW on hypertension can be weakened by a healthy lifestyle [22]. Therefore, maintaining a healthy lifestyle among low-birth-weight children and adolescents might also help to reduce their adverse health effects in current and later life. We also found that children and adolescents in the group of HBW/poor lifestyle had a greater risk than the group of HBW/ideal lifestyle, indicating the need to maintain a healthy lifestyle in children and adolescents with early-life adverse events to avoid cardiovascular metabolism-related diseases. Since the self-reported lifestyle of the latest week could not represent the health effect of long-term lifestyle in children and adolescents and other possible confounding variables, our study only found the association between poor lifestyle and abdominal obesity in children aged 7–12 years, the effect of sleep duration on abdominal obesity and hypertension and the role of screen time on abdominal obesity. Previous longitudinal studies have shown that healthy lifestyle was associated with CMRFs in children and adolescents. Accumulating evidence showed physical activity could affect C-reactive protein and had beneficial effects on the CMRFs in children [63]. In addition, a high-fat diet or deficiencies of specific nutrients could increase the risk of cardiovascular disease [64]. Furthermore, research has shown that screen time and poor sleep were important factors related to cardio-metabolic risk indicators in children [30,65]. However, considering that it is difficult for children and adolescents to achieve a completely healthy lifestyle, our research showed that as long as there were more than two of those healthy lifestyle factors, the risk of clustered CMRFs could be reduced, although significant results were only found in subgroups. This might be due to the small sample size of the subgroups. But prior studies have shown that ideal lifestyle had a positive effect on obesity in children and adolescents with abnormal birth weight [66,67], which could be referred by CMRFs [46].

We also found age and sex differences in clustered CMRFs. The prevalence of clustered CMRFs was higher in adolescents aged 13–18 years than those in children aged 7–12 years, higher in boys than in girls, which was consistent with previous findings [38,68,69,70]. However, a previous longitudinal study showed that birth weight had adverse effects on cardiometabolic metabolism in childhood, but further intensified in adolescence [55], which was not consistent with the conclusions of this study. We found the adverse effect of HBW on clustered CMRFs in the 7–12-year-old group and LBW on hypertension in the 13–18-year-old group, but we did not observe this association in other groups, which indicates that the earlier intervention is conducted, the bigger benefit obtained. Clustered CMRFs in adolescents might mean the track from childhood and the long-term accumulation of adverse outcomes of CMRFs, and simple lifestyle intervention might not work enough, and multiple and fortified measures are needed [27]. In addition, we observed an interesting phenomenon that unhealthy dietary consumption may lead to increased risk of clustered CMRFs in boys but may lead to decreased risk of clustered CMRFs in girls. This may be related to the different weight distribution between sex, with the proportion of overweight and obesity in boys is higher than that in girls, and the proportion of thinness in girls is higher than that in boys [71].

The sample size of this study was obtained from seven provinces in China and could be used as a representative national data. Furthermore, this study was the first one to date addressing the combined effects of birth weight and lifestyle on clustered CMRFs in children and adolescents, providing a strong rationale for promoting cardiovascular and metabolic health in children and adolescents. Moreover, the outcomes measured were based on blood samples, which were more accurate and objective. However, this study still has some limitations. Firstly, our data came from a cross-sectional survey, preventing us from making causal inferences. Secondly, the birth weight and lifestyle factors were collected according to questionnaires and recall bias might exist. However, as important information from a child’s early life, parents were unlikely to forget a child’s birth weight information, and children and adolescents surveyed could recall the lifestyle of the latest week clearly. Thirdly, residual confounding factors existed given that this study did not investigate maternal pre-pregnancy weight, pubertal development in children and adolescents, and other potentially unknown confounding information, which might have some impact on the result. Finally, according to the MCAR test, there were more boys with missing data, which might lead to the underestimation of the results due to higher clustered CMRFs prevalence in boys than girls.

## 5. Conclusions

In summary, we found that the combination of abnormal birth weight and poor lifestyle had a greater impact on clustered CMRFs in children and adolescents. This suggested that avoiding high birth weight through prenatal health education on nutritious diet and exercise guidelines and maintaining a healthy lifestyle during childhood and adolescents or both, such as sufficient physical activity, sleep duration, favorable diet consumption and proper screen time, might reduce the prevalence of CMRFs, especially in younger children.

## Figures and Tables

**Table 1:** Demographic characteristics of eligible children and adolescents and their parents stratified by sex.

Variables	Total	Boys	Girls	χ^2^/F	*p*
Age				8.908	0.003
7–12 years	7090	3609 (63.0)	3481 (60.3)		
13–18 years	4419	2123 (37.0)	2296 (39.7)		
Residence				0.936	0.333
Rural	4523	2278 (39.7)	2245 (38.9)		
Urban	6986	3454 (60.3)	3532 (61.1)		
Single-child status				82.888	<0.001
Yes	7771	4099 (71.5)	3672 (63.6)		
No	3738	1633 (28.5)	2105 (36.4)		
Parental education level				3.841	0.050
Junior high school and below	4499	2292 (40.0)	2207 (38.2)		
Senior high school and above	7010	3440 (60.0)	3570 (61.8)		
Birth weight				58.484	<0.001
NBW	10,044	4913 (85.7)	5131 (88.8)		
LBW	432	191 (3.3)	241 (4.2)		
HBW	1033	628 (11.0)	405 (7.0)		
Fruits intake				1.465	0.226
Inadequate	10,482	5202 (90.8)	5280 (91.4)		
Adequate	1027	530 (9.2)	497 (8.6)		
Vegetables intake				7.865	0.005
Inadequate	10,533	5204 (90.8)	5329 (92.2)		
Adequate	976	528 (9.2)	448 (7.8)		
Meat intake				113.261	<0.001
Improper	9543	4538 (79.2)	5005 (86.6)		
proper	1966	1194 (20.8)	772 (13.4)		
Sugar-sweetened beverage				168.492	<0.001
Excessive	1316	877 (15.3)	439 (7.6)		
Proper	10,193	4855 (84.7)	5338 (92.4)		
Dietary consumption				30.410	<0.001
Unhealthy	8667	4189 (73.1)	4478 (77.5)		
Healthy	2842	1543 (26.9)	1299 (22.5)		
Physical activity				33.352	<0.001
Inadequate	6031	2849 (49.7)	3182 (55.1)		
Adequate	5478	2883 (50.3)	2595 (44.9)		
Screen time				70.682	<0.001
Excessive	3212	1802 (31.4)	1410 (24.4)		
Proper	8297	3930 (68.6)	4367 (75.6)		
Sleep duration				1.951	0.163
Inadequate	9196	4550 (79.4)	4646 (80.4)		
Adequate	2313	1182 (20.6)	1131 (19.6)		
Lifestyle				7.888	0.005
Poor lifestyle	5317	2573 (44.9)	2744 (47.5)		
Ideal lifestyle	6192	3159 (55.1)	3033 (52.5)		
Blood pressure				55.597	<0.001
Normal blood pressure	9478	4568 (79.7)	4910 (85.0)		
Hypertension	2031	1164 (20.3)	867 (15.0)		
Fasting glucoses				39.274	<0.001
Normal fasting glucoses	11,285	5574 (97.2)	5711 (98.9)		
Impaired fasting glucose	224	158 (2.8)	66 (1.1)		
Blood lipids				8.605	0.003
Normal blood lipids	8222	4166 (72.7)	4056 (70.2)		
Dyslipidemia	3287	1566 (27.3)	1721 (29.8)		
Waist circumference				4.408	0.036
Normal waist circumference	8870	4465 (77.9)	4405 (76.3)		
Abdominal obesity	2639	1267 (22.1)	1372 (23.7)		
CMRFs scores					<0.001
0	5725	2868 (50.0)	2857 (49.4)		
1	3815	1825 (31.8)	1990 (34.5)		
2	1553	791 (13.8)	762 (13.2)		
3	406	244 (4.3)	162 (2.8)		
4	10	4 (0.1)	6 (0.1)		
Combinations of birth weight and lifestyle ^a^				13.794	<0.001
NBW/Ideal lifestyle	5430	2720 (47.5)	2710 (46.9)		Ref
NBW/Poor lifestyle	4614	2193 (38.3)	2421 (41.9)		0.050
LBW/Ideal lifestyle	225	109 (1.9)	116 (2.0)		0.992
LBW/Poor lifestyle	207	82 (1.4)	125 (2.2)		0.015
HBW/Ideal lifestyle	537	330 (5.8)	207 (3.6)		<0.001
HBW/Poor lifestyle	496	298 (5.2)	198 (3.4)		<0.001
Total	11,509	5732	5777		

Note: Chi square test was used to compare the two categorical variables. ^a^ ANOVA and Dunnett-*t* test were used to compare the differences between subgroups and NBW/Ideal lifestyle was used as reference group. Abbreviations: NBW, normal birth weight; LBW, low birth weight, HBW, high birth weight, CMRFs, cardio-metabolic risk factors, Ref, the reference group of Dunnett-*t* test.

**Table 2:** Prevalence of CMRFs and clustered CMRFs in different groups.

Variables	Total	Hypertension		Impaired Fasting Glucose		Dyslipidemia		Abdominal Obesity		Clustered CMRFs	
		*n* (%)	*p*	*n* (%)	*p*	*n* (%)	*p*	*n* (%)	*p*	*n* (%)	*p*
Sex			<0.001		<0.001		0.003		0.036		<0.001
Boys	5732	1164 (20.3)		158 (2.8)		1566 (27.3)		1267 (22.1)		248 (4.3)	
Girls	5777	867 (15.0)		66 (1.1)		1721 (29.8)		1372 (23.7)		168 (2.9)	
Age			0.020		<0.001		0.027		0.011		0.022
7–12 years	7090	1205 (17.0)		105 (1.5)		2077 (29.3)		1570 (22.1)		234 (3.3)	
13–18 years	4419	826 (18.7)		119 (2.7)		1210 (27.4)		1069 (24.2)		182 (4.1)	
Residence			<0.001		0.787		<0.001		<0.001		0.331
Rural	4523	963 (21.3)		90 (2.0)		1096 (24.2)		930 (20.6)		173 (3.8)	
Urban	6986	1068 (15.3)		134 (1.9)		2191 (31.4)		1709 (24.5)		243 (3.5)	
Birth weight			0.406		0.297		0.034		<0.001		0.528
NBW	10,044	1756 (17.5)		203 (2.0)		2877 (28.6)		2233 (22.2)		356 (3.5)	
LBW	432	85 (19.7)		7 (1.6)		141 (32.6)		89 (20.6)		19 (4.4)	
HBW	1033	190 (18.4)		14 (1.4)		269 (26.0)		317 (30.7)		41 (4.0)	
Fruits intake			0.428		0.479		0.79		0.293		0.614
Inadequate	10,482	1859 (17.7)		207 (2.0)		2990 (28.5)		2390 (22.8)		376 (3.6)	
Adequate	1027	172 (16.7)		17 (1.7)		297 (28.9)		249 (24.2)		40 (3.9)	
Vegetables intake			0.212		0.029		0.154		0.226		0.897
Inadequate	10,533	1873 (17.8)		214 (2.0)		2989 (28.4)		2400 (22.8)		380 (3.6)	
Adequate	976	158 (16.2)		10 (1.0)		298 (30.5)		239 (24.5)		36 (3.7)	
Meat intake			<0.001		0.397		0.002		0.732		0.017
Improper	9543	1738 (18.2)		181 (1.9)		2781 (29.1)		2194 (23.0)		363 (3.8)	
proper	1966	293 (14.9)		43 (2.2)		506 (25.7)		445 (22.6)		53 (2.7)	
Sugar-sweetened beverage			0.149		0.001		0.117		0.035		0.006
Excessive	1316	251 (19.1)		42 (3.2)		400 (30.4)		332 (25.2)		65 (4.9)	
Proper	10,193	1780 (17.5)		182 (1.8)		2887 (28.3)		2307 (22.6)		351 (3.4)	
Dietary consumption			<0.001		0.193		0.749		0.824		0.312
Unhealthy	8667	1603 (18.5)		177 (2.0)		2482 (28.6)		1983 (22.9)		322 (3.7)	
Healthy	2842	428 (15.1)		47 (1.7)		805 (28.3)		656 (23.1)		94 (3.3)	
Physical activity			0.166		0.724		0.066		0.531		0.424
Inadequate	6031	1036 (17.2)		120 (2.0)		1678 (27.8)		1397 (23.2)		210 (3.5)	
Adequate	5478	995 (18.2)		104 (1.9)		1609 (29.4)		1242 (22.7)		206 (3.8)	
Screen time			0.248		0.326		<0.001		0.415		0.585
Excessive	3212	588 (18.3)		56 (1.7)		845 (26.3)		753 (23.4)		121 (3.8)	
Proper	8297	1443 (17.4)		168 (2.0)		2442 (29.4)		1886 (22.7)		295 (3.6)	
Sleep duration			0.010		0.004		0.075		0.003		0.028
Inadequate	9196	1665 (18.1)		196 (2.1)		2661 (28.9)		2163 (23.5)		350 (3.8)	
Adequate	2313	366 (15.8)		28 (1.2)		626 (27.1)		476 (20.6)		66 (2.9)	
Lifestyle			0.004		0.263		0.002		0.226		0.545
Poor lifestyle	5317	978 (18.4)		113 (2.1)		1474 (27.7)		1258 (23.7)		200 (3.8)	
Ideal lifestyle	6192	1053 (17.0)		111 (1.8)		1813 (29.3)		1381 (22.3)		216 (3.5)	
Combinations of birth weight and lifestyle ^a^			0.277		0.414		0.046		<0.001		0.077
NBW/Ideal lifestyle	5430	921 (17.0)	Ref	102 (1.9)	Ref	1589 (29.3)	Ref	1183 (21.8)	Ref	189 (3.5)	Ref
NBW/Poor lifestyle	4614	835 (18.1)	0.514	101 (2.2)	0.776	1288 (27.9)	0.512	1050 (22.8)	0.754	167 (3.6)	0.998
LBW/Ideal lifestyle	225	41 (18.2)	0.992	2 (0.9)	0.817	80 (35.6)	0.185	43 (19.1)	0.879	13 (5.8)	0.302
LBW/Poor lifestyle	207	44 (21.3)	0.441	5 (2.4)	0.987	61 (29.5)	1.000	46 (22.2)	1.000	6 (2.9)	0.995
HBW/Ideal lifestyle	537	91 (16.9)	1.000	7 (1.3)	0.886	144 (26.8)	0.724	155 (28.9)	0.001	14 (2.6)	0.828
HBW/Poor lifestyle	496	99 (20.0)	0.383	7 (1.4)	0.956	125 (25.2)	0.244	162 (32.7)	<0.001	27 (5.4)	0.117
Total	11,509	2031 (17.6)		224 (1.9)		3287 (28.6)		2639 (22.9)		416 (3.6)	

Note: ^a^ ANOVA and Dunnett-*t* test were used to compare the differences between subgroups and NBW/Ideal lifestyle was used as reference group. Abbreviations: NBW, normal birth weight; LBW, low birth weight, HBW, high birth weight, CMRFs, cardio-metabolic risk factors, Ref, the reference group of Dunnett-*t* test.

**Table 3:** Associations between CMRFs, clustered CMRFs and birth weight stratified by age and sex groups.

	Birth Weight	Hypertension		Impaired Fasting Glucose		Dyslipidemia		Abdominal Obesity		Clustered CMRFs	
		OR (95% CI)	*p*	OR (95% CI)	*p*	OR (95% CI)	*p*	OR (95% CI)	*p*	OR (95% CI)	*p*
Total ^a^	NBW	Ref		Ref		Ref		Ref		Ref	
	LBW	1.21 (0.92–1.60)	0.176	0.99 (0.46–2.17)	0.985	1.13 (0.88–1.44)	0.348	0.98 (0.76–1.27)	0.897	1.43 (0.87–2.36)	0.155
	HBW	1.02 (0.85–1.22)	0.864	0.68 (0.39–1.19)	0.171	0.99 (0.84–1.17)	0.898	1.66 (1.43–1.92)	<0.001	1.18 (0.84–1.67)	0.333
7–12 years ^b^	NBW	Ref		Ref		Ref		Ref		Ref	
	LBW	0.94 (0.64–1.37)	0.737	0.80 (0.25–2.59)	0.706	1.21 (0.89–1.66)	0.218	0.82 (0.58–1.17)	0.281	1.24 (0.62–2.49)	0.541
	HBW	1.08 (0.85–1.36)	0.537	0.60 (0.26–1.41)	0.244	1.06 (0.86–1.30)	0.585	1.74 (1.44–2.10)	<0.001	1.70 (1.12–2.58)	0.013
13–18 years ^b^	NBW	Ref		Ref		Ref		Ref		Ref	
	LBW	1.81 (1.19–2.75)	0.006	1.27 (0.44–3.65)	0.652	0.96 (0.63–1.45)	0.843	1.25 (0.86–1.83)	0.245	1.82 (0.88–3.75)	0.106
	HBW	0.92 (0.69–1.23)	0.567	0.74 (0.35–1.57)	0.434	0.84 (0.63–1.12)	0.235	1.54 (1.21–1.96)	<0.001	0.65 (0.35–1.21)	0.176
Boys ^c^	NBW	Ref		Ref		Ref		Ref		Ref	
	LBW	1.54 (1.06–2.25)	0.024	1.07 (0.42–2.73)	0.883	1.18 (0.82–1.71)	0.369	1.13 (0.77–1.65)	0.534	1.47 (0.75–2.89)	0.257
	HBW	0.98 (0.78–1.23)	0.851	0.81 (0.45–1.48)	0.496	0.98 (0.79–1.22)	0.852	1.48 (1.21–1.81)	<0.001	0.92 (0.58–1.45)	0.713
Girls ^c^	NBW	Ref		Ref		Ref		Ref		Ref	
	LBW	0.96 (0.63–1.46)	0.833	0.95 (0.45–2.01)	0.892	1.07 (0.76–1.49)	0.700	0.87 (0.62–1.23)	0.442	1.44 (0.68–3.03)	0.342
	HBW	1.02 (0.75–1.38)	0.894	0.85 (0.47–1.54)	0.591	0.99 (0.77–1.28)	0.943	1.99 (1.59–2.48)	<0.001	1.83 (1.19–3.08)	0.022

Note: Model ^a^ was adjusted for age, sex, residence, delivery mode, single-child status, parental smoking, parental education, and school effect. Model ^b^ was adjusted for sex, residence, delivery mode, single-child status, parental smoking, parental education, and school effect. Model ^c^ was adjusted for age, residence, delivery mode, single-child status, parental smoking, parental education, and school effect. NBW was used as reference group. NBW, normal birth weight; LBW, low birth weight; HBW, high birth weight; CMRFs, cardio-metabolic risk factors; OR, odds ratio; CI, confidence interval, Ref, the reference group.

**Table 4:** Associations between CMRFs, clustered CMRFs and lifestyles stratified by age and sex group.

	Lifestyles	Hypertension		Impaired Fasting Glucose		Dyslipidemia		Abdominal Obesity		Clustered CMRFs	
		OR (95% CI)	*p*	OR (95% CI)	*p*	OR (95% CI)	*p*	OR (95% CI)	*p*	OR (95% CI)	*p*
Total ^a^	Unhealthy dietary consumption	1.10 (0.97–1.25)	0.132	1.30 (0.93–1.82)	0.129	0.95 (0.85–1.06)	0.360	0.98 (0.88–1.09)	0.695	1.02 (0.80–1.29)	0.905
	Inadequate physical activity	1.01 (0.91–1.12)	0.874	1.06 (0.80–1.40)	0.683	1.00 (0.91–1.10)	0.953	0.99 (0.91–1.09)	0.882	1.01 (0.82–1.24)	0.940
	Excessive screen time	1.04 (0.92–1.17)	0.572	0.99 (0.72–1.36)	0.945	0.98 (0.88–1.09)	0.758	1.17 (1.06–1.30)	0.003	1.16 (0.92–1.45)	0.209
	Inadequate sleep duration	1.18 (1.02–1.36)	0.027	1.11 (0.72–1.71)	0.651	0.97 (0.86–1.10)	0.673	1.14 (1.01–1.29)	0.035	1.17 (0.87–1.56)	0.293
	Poor lifestyle	1.08 (0.97–1.20)	0.181	1.14 (0.86–1.50)	0.368	0.97 (0.88–1.06)	0.500	1.08 (0.99–1.19)	0.085	1.09 (0.89–1.33)	0.431
7–12 years ^b^	Unhealthy dietary consumption	1.00 (0.72–1.37)	0.980	1.51 (0.90–2.54)	0.116	0.94 (0.83–1.08)	0.401	1.02 (0.89–1.17)	0.756	1.02 (0.80–1.29)	0.905
	Inadequate physical activity	1.00 (0.88–1.15)	0.970	0.95 (0.63–1.41)	0.782	1.02 (0.91–1.15)	0.711	1.02 (0.90–1.14)	0.793	1.05 (0.80–1.38)	0.727
	Excessive screen time	1.01 (0.87–1.18)	0.859	1.08 (0.69–1.68)	0.747	0.98 (0.86–1.12)	0.798	1.17 (1.03–1.34)	0.018	1.07 (0.79–1.44)	0.682
	Inadequate sleep duration	1.25 (1.07–1.45)	0.005	1.27 (0.78–2.09)	0.335	0.99 (0.87–1.12)	0.824	1.19 (1.04–1.36)	0.010	1.24 (0.90–1.70)	0.181
	Poor lifestyle	1.12 (0.98–1.29)	0.098	1.05 (0.70–1.58)	0.807	0.95 (0.84–1.07)	0.353	1.15 (1.02–1.29)	0.023	1.03 (0.78–1.35)	0.852
13–18 years ^b^	Unhealthy dietary consumption	1.07 (0.87–1.31)	0.542	1.15 (0.74–1.81)	0.532	0.96 (0.80–1.15)	0.675	0.97 (0.82–1.15)	0.743	1.10 (0.75–1.60)	0.636
	Inadequate physical activity	1.04 (0.88–1.24)	0.639	1.18 (0.79–1.76)	0.417	0.97 (0.83–1.13)	0.665	0.96 (0.83–1.11)	0.600	0.98 (0.71–1.34)	0.879
	Excessive screen time	1.03 (0.85–1.25)	0.764	0.83 (0.51–1.33)	0.429	0.97 (0.81–1.16)	0.708	1.16 (0.99–1.37)	0.075	1.18 (0.83–1.67)	0.364
	Inadequate sleep duration	1.01 (0.68–1.49)	0.973	0.75 (0.31–1.83)	0.532	0.95 (0.65–1.38)	0.776	0.96 (0.68–1.37)	0.822	1.10 (0.54–2.27)	0.792
	Poor lifestyle	1.03 (0.87–1.22)	0.721	1.19 (0.81–1.75)	0.384	1.01 (0.86–1.18)	0.937	1.02 (0.88–1.18)	0.784	1.17 (0.85–1.60)	0.338
Boys ^c^	Unhealthy dietary consumption	1.11 (0.94–1.31)	0.213	1.31 (0.88–1.96)	0.184	1.00 (0.86–1.16)	0.999	1.03 (0.89–1.20)	0.667	1.40 (1.01–1.94)	0.046
	Inadequate physical activity	1.00 (0.87–1.15)	0.988	1.07 (0.76–1.49)	0.704	0.95 (0.83–1.09)	0.422	0.96 (0.84–1.10)	0.551	1.12 (0.86–1.46)	0.411
	Excessive screen time	1.05 (0.90–1.23)	0.507	1.06 (0.73–1.53)	0.769	0.94 (0.81–1.09)	0.435	1.17 (1.01–1.35)	0.034	1.10 (0.83–1.48)	0.504
	Inadequate sleep duration	1.12 (0.93–1.36)	0.236	1.08 (0.65–1.79)	0.764	1.05 (0.88–1.25)	0.613	1.15 (0.97–1.37)	0.113	1.20 (0.81–1.76)	0.369
	Poor lifestyle	1.08 (0.93–1.24)	0.304	1.22 (0.87–1.70)	0.244	0.97 (0.85–1.11)	0.677	1.09 (0.95–1.24)	0.213	1.28 (0.98–1.67)	0.070
Girls ^c^	Unhealthy dietary consumption	1.11 (0.91–1.35)	0.304	1.04 (0.74–1.47)	0.817	0.90 (0.77–1.05)	0.192	0.96 (0.82–1.11)	0.551	0.68 (0.47–0.97)	0.034
	Inadequate physical activity	1.02 (0.87–1.20)	0.803	0.99 (0.74–1.32)	0.929	1.04 (0.91–1.19)	0.558	1.02 (0.89–1.16)	0.801	0.85 (0.62–1.17)	0.327
	Excessive screen time	1.00 (0.83–1.21)	0.973	0.93 (0.66–1.31)	0.676	1.03 (0.88–1.20)	0.738	1.18 (1.02–1.36)	0.031	1.21 (0.84–1.74)	0.313
	Inadequate sleep duration	1.28 (1.03–1.59)	0.028	1.06 (0.71–1.57)	0.789	0.91 (0.77–1.08)	0.270	1.11 (0.93–1.32)	0.263	1.18 (0.76–1.82)	0.456
	Poor lifestyle	1.09 (0.93–1.28)	0.304	0.97 (0.73–1.30)	0.857	0.95 (0.84–1.09)	0.486	1.08 (0.95–1.23)	0.237	0.85 (0.61–1.17)	0.323

Note: Model ^a^ was adjusted for age, sex, residence, delivery mode, single-child status, parental smoking, parental education, the interaction of sex and birth weight and school effect. Model ^b^ was adjusted for sex, residence, delivery mode, single-child status, parental smoking, parental education, and school effect. Model ^c^ was adjusted for age, residence, delivery mode, single-child status, parental smoking, parental education, and school effect. Healthy dietary consumption, adequate physical activity, proper screen time and adequate sleep duration were used as reference groups. NBW, normal birth weight; LBW, low birth weight; HBW, high birth weight; CMRFs, cardio-metabolic risk factors; OR, odds ratio; CI, confidence interval.

**Table 5:** Associations between CMRFs, clustered CMRFs and combinations of birth weight and lifestyle in mixed effect models stratified by age and sex groups.

	Combinations	Hypertension		Impaired Fasting Glucose		Dyslipidemia		Abdominal Obesity		Clustered CMRFs	
		OR (95% CI)	*p*	OR (95% CI)	*p*	OR (95% CI)	*p*	OR (95% CI)	*p*	OR (95% CI)	*p*
Total ^a^	NBW/Ideal lifestyle	Ref		Ref		Ref		Ref *		Ref	
	NBW/Poor lifestyle	1.06 (0.95–1.19)	0.302	1.12 (0.83–1.49)	0.459	0.98 (0.89–1.08)	0.683	1.06 (0.96–1.17) *	0.237	1.04 (0.83–1.29)	0.754
	LBW/Ideal lifestyle	1.25 (0.84–1.85)	0.269	0.56 (0.14–2.34)	0.428	1.35 (0.97–1.88)	0.074	0.94 (0.66–1.36)	0.759	2.00 (1.07–3.72)	0.029
	LBW/Poor lifestyle	1.25 (0.84–1.84)	0.274	1.59 (0.62–4.10)	0.333	0.89 (0.61–1.29)	0.540	1.08 (0.76–1.54)	0.669	0.95 (0.41–2.19)	0.896
	HBW/Ideal lifestyle	0.94 (0.73–1.22)	0.652	0.70 (0.32–1.55)	0.384	0.98 (0.78–1.22)	0.841	1.54 (1.26–1.90)	<0.001	0.76 (0.43–1.33)	0.327
	HBW/Poor lifestyle	1.16 (0.90–1.49)	0.249	0.72 (0.33–1.58)	0.411	0.99 (0.78–1.25)	0.898	1.89 (1.53–2.33)	<0.001	1.74 (1.13–2.68)	0.012
7–12 years ^b^	NBW/Ideal lifestyle	Ref		Ref		Ref		Ref *		Ref	
	NBW/Poor lifestyle	1.11 (0.96–1.29)	0.159	1.12 (0.74–1.71)	0.592	0.92 (0.81–1.05)	0.222	1.10 (0.97–1.25) *	0.134	0.89 (0.66–1.21)	0.470
	LBW/Ideal lifestyle	0.83 (0.49–1.43)	0.504	1.03 (0.24–4.37)	0.974	1.21 (0.80–1.83)	0.367	0.74 (0.45–1.21)	0.228	1.02 (0.36–2.84)	0.977
	LBW/Poor lifestyle	1.16 (0.68–1.99)	0.580	0.61 (0.08–4.59)	0.632	1.14 (0.72–1.80)	0.583	1.01 (0.62–1.66)	0.963	1.38 (0.54–3.53)	0.506
	HBW/Ideal lifestyle	1.06 (0.78–1.44)	0.720	0.79 (0.28–2.24)	0.653	0.97 (0.74–1.27)	0.798	1.55 (1.20–2.00) *	0.001	1.07 (0.56–2.04)	0.838
	HBW/Poor lifestyle	1.21 (0.86–1.72)	0.273	0.46 (0.11–1.92)	0.284	1.11 (0.82–1.49)	0.508	2.20 (1.67–2.88)	<0.001	2.37 (1.39–4.05)	0.002
13–18 years ^b^	NBW/Ideal lifestyle	Ref		Ref		Ref		Ref *		Ref	
	NBW/Poor lifestyle	1.10 (0.86–1.39)	0.458	1.26 (0.78–2.05)	0.343	1.07 (0.87–1.31)	0.550	0.90 (0.74–1.10) *	0.316	1.18 (0.77–1.81)	0.442
	LBW/Ideal lifestyle	2.43 (1.15–5.17)	0.021	1.00 (0.66–1.62)	0.981	1.69 (0.86–3.31) #	0.129	1.38 (0.72–2.65)	0.338	3.57 (1.23–10.37)	0.019
	LBW/Poor lifestyle	1.32 (0.58–2.99)	0.506	2.03 (0.44–9.43)	0.366	0.51 (0.21–1.24)	0.137	0.98 (0.49–1.96)	0.950	1.00 (0.62–1.48)	0.970
	HBW/Ideal lifestyle	0.64 (0.34–1.19)	0.157	1.00 (0.64–1.52)	0.970	1.04 (0.64–1.69)	0.879	1.76 (1.16–2.67)	0.008	0.36 (0.08–1.58)	0.177
	HBW/Poor lifestyle	1.32 (0.78–2.23)	0.296	1.58 (0.58–4.34)	0.372	0.64 (0.35–1.16)	0.139	1.70 (1.09–2.65)	0.019	1.06 (0.40–2.83)	0.907
Boys ^c^	NBW/Ideal lifestyle	Ref		Ref		Ref		Ref *		Ref	
	NBW/Poor lifestyle	1.15 (0.92–1.43)	0.215	1.65 (1.02–2.67)	0.043	1.03 (0.85–1.24)	0.757	1.07 (0.90–1.28) *	0.451	1.33 (0.91–1.95)	0.135
	LBW/Ideal lifestyle	2.45 (1.25–4.79)	0.009	0.92 (0.12–7.10)	0.935	1.14 (0.61–2.12)	0.682	1.03 (0.55–1.95)	0.920	2.78 (1.02–7.62)	0.047
	LBW/Poor lifestyle	1.36 (0.64–2.91)	0.428	1.02 (0.13–7.99)	0.988	0.68 (0.31–1.50)	0.342	1.08 (0.54–2.17)	0.829	0.56 (0.08–4.23)	0.576
	HBW/Ideal lifestyle	0.93 (0.58–1.50)	0.775	0.31 (0.04–2.31)	0.251	1.31 (0.90–1.90)	0.155	1.56 (1.10–2.19)	0.012	0.76 (0.29–1.98)	0.574
	HBW/Poor lifestyle	1.03 (0.63–1.67)	0.911	2.03 (0.79–5.21)	0.139	0.88 (0.57–1.37)	0.569	2.49 (1.73–3.57)	<0.001	1.19 (0.51–2.74)	0.689
Girls ^c^	NBW/Ideal lifestyle	Ref		Ref		Ref		Ref *		Ref	
	NBW/Poor lifestyle	1.01 (0.79–1.29)	0.928	1.00 (0.68–1.49)	0.991	0.98 (0.81–1.17)	0.788	0.96 (0.81–1.15) *	0.689	0.68 (0.41–1.13) *	0.135
	LBW/Ideal lifestyle	1.24 (0.59–2.62)	0.572	0.80 (0.20–3.30)	0.758	1.40 (0.80–2.46)	0.244	0.91 (0.50–1.68)	0.771	1.60 (0.46–5.55)	0.462
	LBW/Poor lifestyle	1.36 (0.58–3.20)	0.477	1.04 (0.27–4.08)	0.956	1.12 (0.59–2.15)	0.723	0.90 (0.47–1.73)	0.753	1.19 (0.26–5.41)	0.818
	HBW/Ideal lifestyle	0.86 (0.48–1.54)	0.600	0.91 (0.33–2.47)	0.848	1.06 (0.68–1.65)	0.789	2.00 (1.37–2.93)	<0.001	1.38 (0.52–3.68)	0.520
	HBW/Poor lifestyle	1.53 (0.86–2.72)	0.151	0.80 (0.24–2.70)	0.715	1.02 (0.61–1.70)	0.950	2.08 (1.34–3.20)	0.001	1.92 (0.72–5.10)	0.191

Note: Model ^a^ was adjusted for age, sex, residence, delivery mode, single-child status, parental smoking, parental education, and school effect. Model ^b^ was adjusted for sex, residence, delivery mode, single-child status, parental smoking, parental education, and school effect. Model ^c^ was adjusted for age, residence, delivery mode, single-child status, parental smoking, parental education, and school effect. NBW/Ideal lifestyle was used as reference group. NBW, normal birth weight; LBW, low birth weight; HBW, high birth weight; CMRFs, cardio-metabolic risk factors; OR, odds ratio; CI, confidence interval, Ref, the reference group. #, the odds ratio difference from the group of LBW/Poor lifestyle was statistically significant, LBW/Poor lifestyle was used as reference group. * The odds ratio difference from the group of HBW/Poor lifestyle was statistically significant, HBW/Poor lifestyle was used as reference group.

## Data Availability

The data supporting the conclusions of this article can be made available from the corresponding author upon request.

## Supplementary Materials

The following supporting information can be downloaded at: https://www.mdpi.com/article/10.3390/nu14153131/s1, Table S1: Missing completely at random (MCAR) test of the sample. Figure S1: Associations between hypertension, impaired fasting glucose and birth weight stratified by age and sex groups; Figure S2: Associations between dyslipidemia, abdominal obesity and birth weight stratified by age and sex groups; Figure S3: Associations between clustered CMRFs and birth weight stratified by age and sex groups; Figure S4: Associations between hypertension, impaired fasting glucose and lifestyle stratified by age and sex groups; Figure S5: Associations between dyslipidemia, abdominal obesity and lifestyle stratified by age and sex groups; Figure S6: Associations between clustered CMRFs and lifestyle stratified by age and sex groups; Figure S7: Associations between hypertension, impaired fasting glucose and combinations of birth weight and lifestyle stratified by age and sex groups; Figure S8: Associations between dyslipidemia, abdominal obesity and combinations of birth weight and lifestyle stratified by age and sex groups; Figure S9: Associations between clustered CMRFs and combinations of birth weight and lifestyle stratified by age and sex groups.
