# Supplementary material for: The Combined Effect of Birth Weight and Lifestyle on Clustered Cardio-Metabolic Risk Factors in Children and Adolescents: A National School-Based Cross-Sectional Survey
Source: Nutrients. 2022 Jul 29;14(15):3131. doi: 10.3390/nu14153131 (PMC9370142; doi:10.3390/nu14153131)
Supplement: Supplementary file 1 [file nutrients-14-03131-s001.zip › nutrients-1830575-supplementary.pdf]

Table S1. Missing completely at random (MCAR) test of the sample

	**N of primary sample**	**N of final sample**	χ^2	***P*-value**
Age (years)	11.43	11.36		0.092
Sex			6.864	0.009
Boys	8088 （51.4）	5732 （49.8）		
Girls	7644 （48.6）	5777 （50.2）		
Residence			0.916	0.338
Rural	5545 （41.0）	4523 （40.4）		
Urban	7964 （59.0）	6660 （59.6）		
Parental education level			0.136	0.712
Senior high school and above	5798 （44.4）	4930 （44.1）		
Junior high school and below	7271 （55.6）	6242 （55.9）		

	Birth weight	Hypertension OR(95%CI)		*P*	Impaired fasting glucose OR(95%CI)		*P*
Total [a]	NBW						
	LBW	1.21(0.92-1.60)		0.176	0.99(0.46-2.17)		0.985
	HBW	1.02(0.85-1.22)		0.864	0.68(0.39-1.19)		0.171
7-12 years [b]	NBW						
	LBW	0.94(0.64-1.37)		0.737	0.80(0.25-2.59)		0.706
	HBW	1.08(0.85-1.36)		0.537	0.60(0.26-1.41)		0.244
13-18 years [b]	NBW						
	LBW	1.81(1.19-2.75)		0.006	1.27(0.44-3.65)		0.652
	HBW	0.92(0.69-1.23)		0.567	0.74(0.35-1.57)		0.434
Boys [c]	NBW						
	LBW	1.54(1.06-2.25)		0.024	1.07(0.42-2.73)		0.883
	HBW	0.98(0.78-1.23)		0.851	0.81(0.45-1.48)		0.496
Girls [c]	NBW						
	LBW	0.96(0.63-1.46)		0.833	0.95(0.45-2.01)		0.892
	HBW	1.02(0.75-1.38)		0.894	0.85(0.47-1.54)		0.591

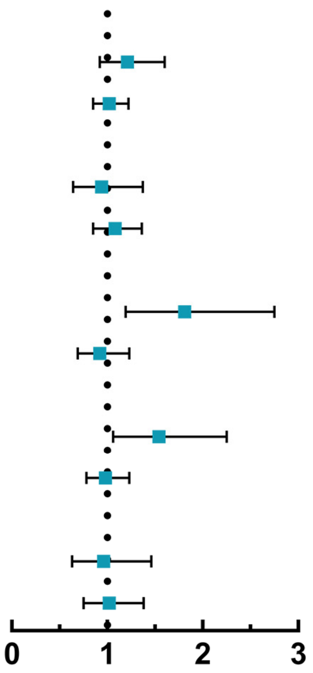

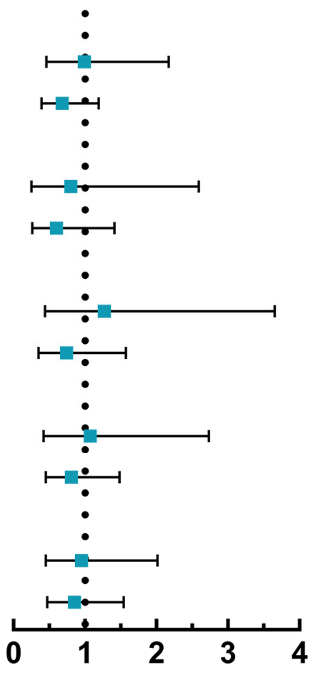

Figure S1. Associations between hypertension, impaired fasting glucose and birth weight stratified by age and sex groups

Note: Model a was adjusted for age, sex, residence, delivery mode, single-child status, parental smoking, parental education and school effect. Model b was adjusted for sex, residence, delivery mode, single-child status, parental smoking, parental education and school effect. Model c was adjusted for age, residence, delivery mode, single-child status, parental smoking, parental education and school effect. NBW, normal birth weight; LBW, low birth weight; HBW, high birth weight; CMRFs, cardio-metabolic risk factors; OR, odds ratio; CI, confidence interval.

	Birth weight	Dyslipidemia OR(95%CI)	Dyslipidemia *P*	Abdominal obesity OR(95%CI)	Abdominal obesity *P*
Total [a]	NBW				
	LBW	1.13(0.88-1.44)	0.348	0.98(0.76-1.27)	0.897
	HBW	0.99(0.84-1.17)	0.898	1.66(1.43-1.92)	< 0.001
7-12 years [b]	NBW				
	LBW	1.21(0.89-1.66)	0.218	0.82(0.58-1.17)	0.281
	HBW	1.06(0.86-1.30)	0.585	1.74(1.44-2.10)	< 0.001
13-18 years [b]	NBW				
	LBW	0.96(0.63-1.45)	0.843	1.25(0.86-1.83)	0.245
	HBW	0.84(0.63-1.12)	0.235	1.54(1.21-1.96)	< 0.001
Boys [c]	NBW				
	LBW	1.18(0.82-1.71)	0.369	1.13(0.77-1.65)	0.534
	HBW	0.98(0.79-1.22)	0.852	1.48(1.21-1.81)	< 0.001
Girls [c]	NBW				
	LBW	1.07(0.76-1.49)	0.700	0.87(0.62-1.23)	0.442
	HBW	0.99(0.77-1.28)	0.943	1.99(1.59-2.48)	< 0.001

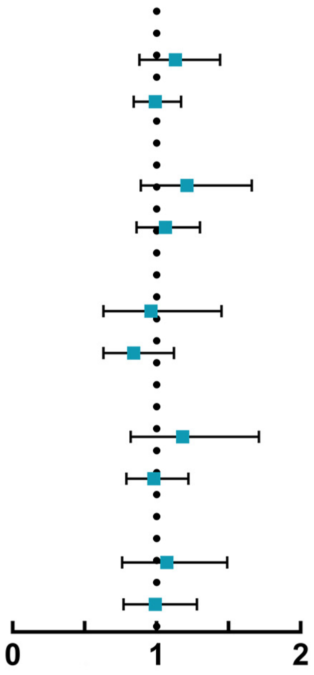

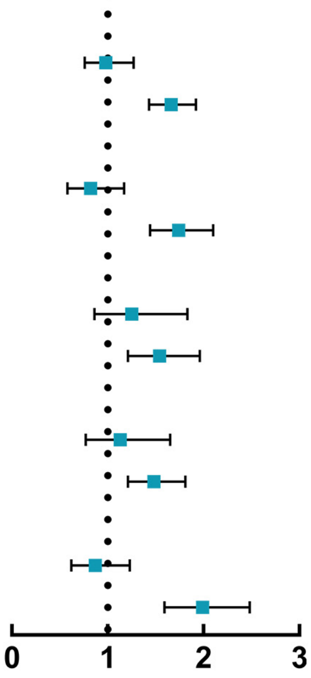

Figure S2. Associations between dyslipidemia, abdominal obesity and birth weight stratified by age and sex groups

Note: Model a was adjusted for age, sex, residence, delivery mode, single-child status, parental smoking, parental education and school effect. Model b was adjusted for sex, residence, delivery mode, single-child status, parental smoking, parental education and school effect. Model c was adjusted for age, residence, delivery mode, single-child status, parental smoking, parental education and school effect. NBW, normal birth weight; LBW, low birth weight; HBW, high birth weight; CMRFs, cardio-metabolic risk factors; OR, odds ratio; CI, confidence interval.

	Birth weight	Clustered CMRFs OR(95%CI)	*P*
Total [a]	NBW		
	LBW	1.43(0.87-2.36)	0.155
	HBW	1.18(0.84-1.67)	0.333
7-12 years [b]	NBW		
	LBW	1.24(0.62-2.49)	0.541
	HBW	1.70(1.12-2.58)	0.013
13-18 years [b]	NBW		
	LBW	1.82(0.88-3.75)	0.106
	HBW	0.65(0.35-1.21)	0.176
Boys [c]	NBW		
	LBW	1.47(0.75-2.89)	0.257
	HBW	0.92(0.58-1.45)	0.713
Girls [c]	NBW		
	LBW	1.44(0.68-3.03)	0.342
	HBW	1.83(1.19-3.08)	0.022

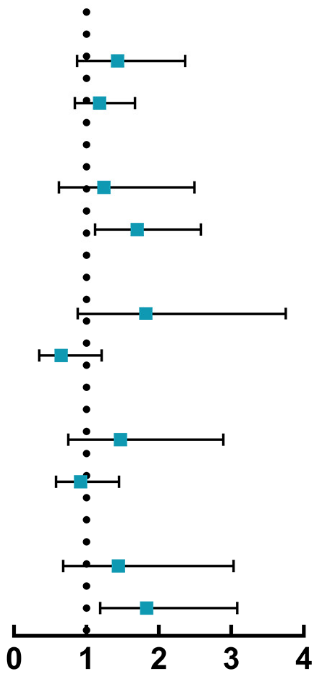

Figure S3. Associations between clustered CMRFs and birth weight stratified by age and sex groups

Note: Model a was adjusted for age, sex, residence, delivery mode, single-child status, parental smoking, parental education and school effect. Model b was adjusted for sex, residence, delivery mode, single-child status, parental smoking, parental education and school effect. Model c was adjusted for age, residence, delivery mode, single-child status, parental smoking, parental education and school effect. NBW, normal birth weight; LBW, low birth weight; HBW, high birth weight; CMRFs, cardio-metabolic risk factors; OR, odds ratio; CI, confidence interval.

Lifestyles		Hypertension OR (95%CI)	Hypertension *P*	Impaired fasting glucose OR (95%CI)	Impaired fasting glucose *P*
Total [a]	Unhealthy dietary consumption	1.10(0.97-1.25)	0.132	1.30(0.93-1.82)	0.129
	Inadequate physical activity	1.01(0.91-1.12)	0.874	1.06(0.80-1.40)	0.683
	Excessive screen time	1.04(0.92-1.17)	0.572	0.99(0.72-1.36)	0.945
	Inadequate sleep duration	1.18(1.02-1.36)	0.027	1.11(0.72-1.71)	0.651
	Poor lifestyle	1.08(0.97-1.20)	0.181	1.14(0.86-1.50)	0.368
7-12 years [b]	Unhealthy dietary consumption	1.00(0.72-1.37)	0.980	1.51(0.90-2.54)	0.116
	Inadequate physical activity	1.00(0.88-1.15)	0.970	0.95(0.63-1.41)	0.782
	Excessive screen time	1.01(0.87-1.18)	0.859	1.08(0.69-1.68)	0.747
	Inadequate sleep duration	1.25(1.07-1.45)	0.005	1.27(0.78-2.09)	0.335
	Poor lifestyle	1.12(0.98-1.29)	0.098	1.05(0.70-1.58)	0.807
13-18 years [b]	Unhealthy dietary consumption	1.07(0.87-1.31)	0.542	1.15(0.74-1.81)	0.532
	Inadequate physical activity	1.04(0.88-1.24)	0.639	1.18(0.79-1.76)	0.417
	Excessive screen time	1.03(0.85-1.25)	0.764	0.83(0.51-1.33)	0.429
	Inadequate sleep duration	1.01(0.68-1.49)	0.973	0.75(0.31-1.83)	0.532
	Poor lifestyle	1.03(0.87-1.22)	0.721	1.19(0.81-1.75)	0.384
Boys [c]	Unhealthy dietary consumption	1.11(0.94-1.31)	0.213	1.31(0.88-1.96)	0.184
	Inadequate physical activity	1.00(0.87-1.15)	0.988	1.07(0.76-1.49)	0.704
	Excessive screen time	1.05(0.90-1.23)	0.507	1.06(0.73-1.53)	0.769
	Inadequate sleep duration	1.12(0.93-1.36)	0.236	1.08(0.65-1.79)	0.764
	Poor lifestyle	1.08(0.93-1.24)	0.304	1.22(0.87-1.70)	0.244
Girls [c]	Unhealthy dietary consumption	1.11(0.91-1.35)	0.304	1.04(0.74-1.47)	0.817
	Inadequate physical activity	1.02(0.87-1.20)	0.803	0.99(0.74-1.32)	0.929
	Excessive screen time	1.00(0.83-1.21)	0.973	0.93(0.66-1.31)	0.676
	Inadequate sleep duration	1.28(1.03-1.59)	0.028	1.06(0.71-1.57)	0.789
	Poor lifestyle	1.09(0.93-1.28)	0.304	0.97(0.73-1.30)	0.857

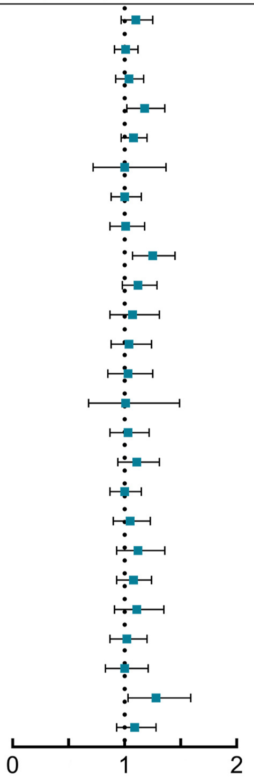

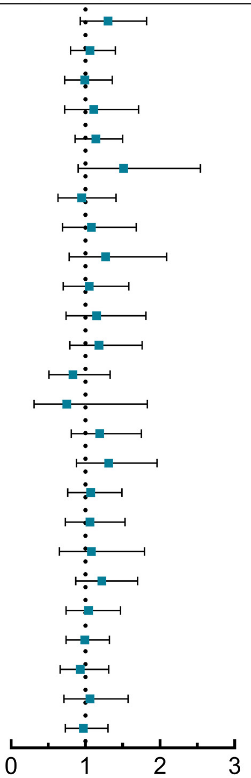

Figure S4. Associations between hypertension, impaired fasting glucose and lifestyle stratified by age and sex groups

Note: Model a was adjusted for age, sex, residence, delivery mode, single-child status, parental smoking, parental education and school effect. Model b was adjusted for sex, residence, delivery mode, single-child status, parental smoking, parental education and school effect. Model c was adjusted for age, residence, delivery mode, single-child status, parental smoking, parental education and school effect. NBW, normal birth weight; LBW, low birth weight; HBW, high birth weight; CMRFs, cardio-metabolic risk factors; OR, odds ratio; CI, confidence interval.

Lifestyles		Dyslipidemia		Abdominal obesity	
		OR (95%CI)	*P*	OR (95%CI)	*P*
Total [a]	Unhealthy dietary consumption	0.95(0.85-1.06)	0.360	0.98(0.88-1.09)	0.695
	Inadequate physical activity	1.00(0.91-1.10)	0.953	0.99(0.91-1.09)	0.882
	Excessive screen time	0.98(0.88-1.09)	0.758	1.17(1.06-1.30)	0.003
	Inadequate sleep duration	0.97(0.86-1.10)	0.673	1.14(1.01-1.29)	0.035
	Poor lifestyle	0.97(0.88-1.06)	0.500	1.08(0.99-1.19)	0.085
7-12 years [b]	Unhealthy dietary consumption	0.94(0.83-1.08)	0.401	1.02(0.89-1.17)	0.756
	Inadequate physical activity	1.02(0.91-1.15)	0.711	1.02(0.90-1.14)	0.793
	Excessive screen time	0.98(0.86-1.12)	0.798	1.17(1.03-1.34)	0.018
	Inadequate sleep duration	0.99(0.87-1.12)	0.824	1.19(1.04-1.36)	0.010
	Poor lifestyle	0.95(0.84-1.07)	0.353	1.15(1.02-1.29)	0.023
13-18 years [b]	Unhealthy dietary consumption	0.96(0.80-1.15)	0.675	0.97(0.82-1.15)	0.743
	Inadequate physical activity	0.97(0.83-1.13)	0.665	0.96(0.83-1.11)	0.600
	Excessive screen time	0.97(0.81-1.16)	0.708	1.16(0.99-1.37)	0.075
	Inadequate sleep duration	0.95(0.65-1.38)	0.776	0.96(0.68-1.37)	0.822
	Poor lifestyle	1.01(0.86-1.18)	0.937	1.02(0.88-1.18)	0.784
Boys [c]	Unhealthy dietary consumption	1.00(0.86-1.16)	0.999	1.03(0.89-1.20)	0.667
	Inadequate physical activity	0.95(0.83-1.09)	0.422	0.96(0.84-1.10)	0.551
	Excessive screen time	0.94(0.81-1.09)	0.435	1.17(1.01-1.35)	0.034
	Inadequate sleep duration	1.05(0.88-1.25)	0.613	1.15(0.97-1.37)	0.113
	Poor lifestyle	0.97(0.85-1.11)	0.677	1.09(0.95-1.24)	0.213
Girls [c]	Unhealthy dietary consumption	0.90(0.77-1.05)	0.192	0.96(0.82-1.11)	0.551
	Inadequate physical activity	1.04(0.91-1.19)	0.558	1.02(0.89-1.16)	0.801
	Excessive screen time	1.03(0.88-1.20)	0.738	1.18(1.02-1.36)	0.031
	Inadequate sleep duration	0.91(0.77-1.08)	0.270	1.11(0.93-1.32)	0.263
	Poor lifestyle	0.95(0.84-1.09)	0.486	1.08(0.95-1.23)	0.237

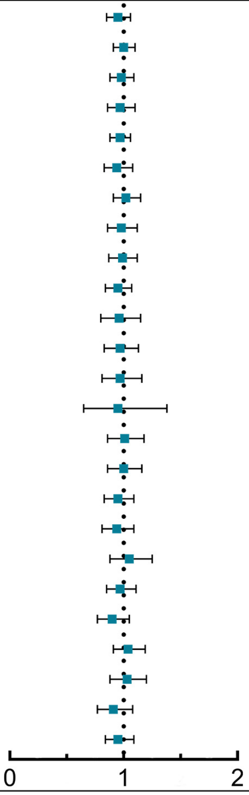

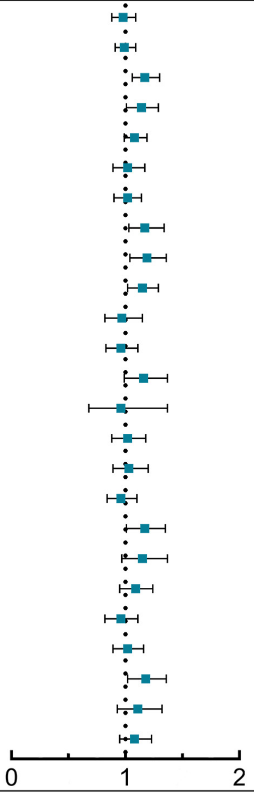

Figure S5. Associations between dyslipidemia, abdominal obesity and lifestyle stratified by age and sex groups

Note: Model a was adjusted for age, sex, residence, delivery mode, single-child status, parental smoking, parental education and school effect. Model b was adjusted for sex, residence, delivery mode, single-child status, parental smoking, parental education and school effect. Model c was adjusted for age, residence, delivery mode, single-child status, parental smoking, parental education and school effect. NBW, normal birth weight; LBW, low birth weight; HBW, high birth weight; CMRFs, cardio-metabolic risk factors; OR, odds ratio; CI, confidence interval.

Lifestyles		Clustered CMRFs OR (95%CI)	P
Total [a]	Unhealthy dietary consumption	1.02(0.80-1.29)	0.905
	Inadequate physical activity	1.01(0.82-1.24)	0.940
	Excessive screen time	1.16(0.92-1.45)	0.209
	Inadequate sleep duration	1.17(0.87-1.56)	0.293
	Poor lifestyle	1.09(0.89-1.33)	0.431
7-12 years [b]	Unhealthy dietary consumption	1.02(0.80-1.29)	0.905
	Inadequate physical activity	1.05(0.80-1.38)	0.727
	Excessive screen time	1.07(0.79-1.44)	0.682
	Inadequate sleep duration	1.24(0.90-1.70)	0.181
	Poor lifestyle	1.03(0.78-1.35)	0.852
13-18 years [b]	Unhealthy dietary consumption	1.10(0.75-1.60)	0.636
	Inadequate physical activity	0.98(0.71-1.34)	0.879
	Excessive screen time	1.18(0.83-1.67)	0.364
	Inadequate sleep duration	1.10(0.54-2.27)	0.792
	Poor lifestyle	1.17(0.85-1.60)	0.338
Boys [c]	Unhealthy dietary consumption	1.40(1.01-1.94)	0.046
	Inadequate physical activity	1.12(0.86-1.46)	0.411
	Excessive screen time	1.10(0.83-1.48)	0.504
	Inadequate sleep duration	1.20(0.81-1.76)	0.369
	Poor lifestyle	1.28(0.98-1.67)	0.070
Girls [c]	Unhealthy dietary consumption	0.68(0.47-0.97)	0.034
	Inadequate physical activity	0.85(0.62-1.17)	0.327
	Excessive screen time	1.21(0.84-1.74)	0.313
	Inadequate sleep duration	1.18(0.76-1.82)	0.456
	Poor lifestyle	0.85(0.61-1.17)	0.323

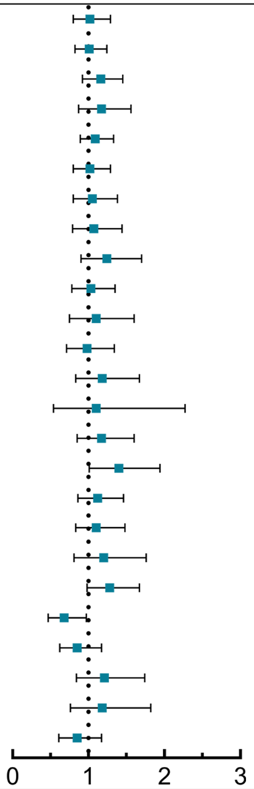

Figure S6. Associations between clustered CMRFs and lifestyle stratified by age and sex groups

Note: Model a was adjusted for age, sex, residence, delivery mode, single-child status, parental smoking, parental education and school effect. Model b was adjusted for sex, residence, delivery mode, single-child status, parental smoking, parental education and school effect. Model c was adjusted for age, residence, delivery mode, single-child status, parental smoking, parental education and school effect. NBW, normal birth weight; LBW, low birth weight; HBW, high birth weight; CMRFs, cardio-metabolic risk factors; OR, odds ratio; CI, confidence interval.

	Combinations	Hypertension OR (95%CI)	Hypertension *P*	Impaired fasting glucose OR (95%CI)	Impaired fasting glucose *P*
Total [a]	NBW/Ideal lifestyle				
	NBW/Poor lifestyle	1.06(0.95-1.19)	0.302	1.12(0.83-1.49)	0.459
	LBW/Ideal lifestyle	1.25(0.84-1.85)	0.269	0.56(0.14-2.34)	0.428
	LBW/Poor lifestyle	1.25(0.84-1.84)	0.274	1.59(0.62-4.10)	0.333
	HBW/Ideal lifestyle	0.94(0.73-1.22)	0.652	0.70(0.32-1.55)	0.384
	HBW/Poor lifestyle	1.16(0.90-1.49)	0.249	0.72(0.33-1.58)	0.411
7-12 years [b]	NBW/Ideal lifestyle				
	NBW/Poor lifestyle	1.11(0.96-1.29)	0.159	1.12(0.74-1.71)	0.592
	LBW/Ideal lifestyle	0.83(0.49-1.43)	0.504	1.03(0.24-4.37)	0.974
	LBW/Poor lifestyle	1.16(0.68-1.99)	0.580	0.61(0.08-4.59)	0.632
	HBW/Ideal lifestyle	1.06(0.78-1.44)	0.720	0.79(0.28-2.24)	0.653
	HBW/Poor lifestyle	1.21(0.86-1.72)	0.273	0.46(0.11-1.92)	0.284
13-18 years [b]	NBW/Ideal lifestyle				
	NBW/Poor lifestyle	1.10(0.86-1.39)	0.458	1.26(0.78-2.05)	0.343
	LBW/Ideal lifestyle	2.43(1.15-5.17)	0.021	1.00(0.66-1.62)	0.981
	LBW/Poor lifestyle	1.32(0.58-2.99)	0.506	2.03(0.44-9.43)	0.366
	HBW/Ideal lifestyle	0.64(0.34-1.19)	0.157	1.00(0.64-1.52)	0.970
	HBW/Poor lifestyle	1.32(0.78-2.23)	0.296	1.58(0.58-4.34)	0.372
Boys [c]	NBW/Ideal lifestyle				
	NBW/Poor lifestyle	1.15(0.92-1.43)	0.215	1.65(1.02-2.67)	0.043
	LBW/Ideal lifestyle	2.45(1.25-4.79)	0.009	0.92(0.12-7.10)	0.935
	LBW/Poor lifestyle	1.36(0.64-2.91)	0.428	1.02(0.13-7.99)	0.988
	HBW/Ideal lifestyle	0.93(0.58-1.50)	0.775	0.31(0.04-2.31)	0.251
	HBW/Poor lifestyle	1.03(0.63-1.67)	0.911	2.03(0.79-5.21)	0.139
Girls [c]	NBW/Ideal lifestyle				
	NBW/Poor lifestyle	1.01(0.79-1.29)	0.928	1.00(0.68-1.49)	0.991
	LBW/Ideal lifestyle	1.24(0.59-2.62)	0.572	0.80(0.20-3.30)	0.758
	LBW/Poor lifestyle	1.36(0.58-3.20)	0.477	1.04(0.27-4.08)	0.956
	HBW/Ideal lifestyle	0.86(0.48-1.54)	0.600	0.91(0.33-2.47)	0.848
	HBW/Poor lifestyle	1.53(0.86-2.72)	0.151	0.80(0.24-2.70)	0.715

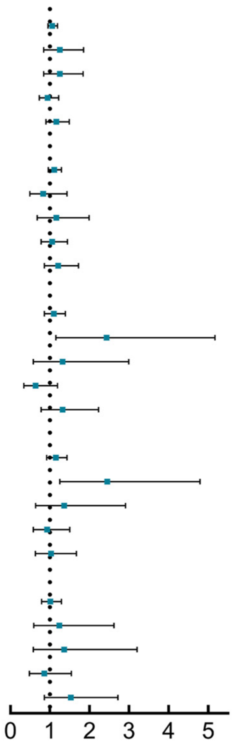

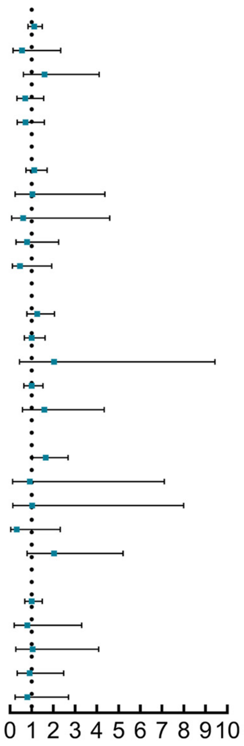

Figure S7. Associations between hypertension, impaired fasting glucose and combinations of birth weight and lifestyle stratified by age and sex groups

Note: Model a was adjusted for age, sex, residence, delivery mode, single-child status, parental smoking, parental education and school effect. Model b was adjusted for sex, residence, delivery mode, single-child status, parental smoking, parental education and school effect. Model c was adjusted for age, residence, delivery mode, single-child status, parental smoking, parental education and school effect. NBW, normal birth weight; LBW, low birth weight; HBW, high birth weight; CMRFs, cardio-metabolic risk factors; OR, odds ratio; CI, confidence interval. #, the odds ratio was differed from the group of LBW/Poor lifestyle statistically significant. *, the odds ratio was differed from the group of HBW/Poor lifestyle statistically significant.

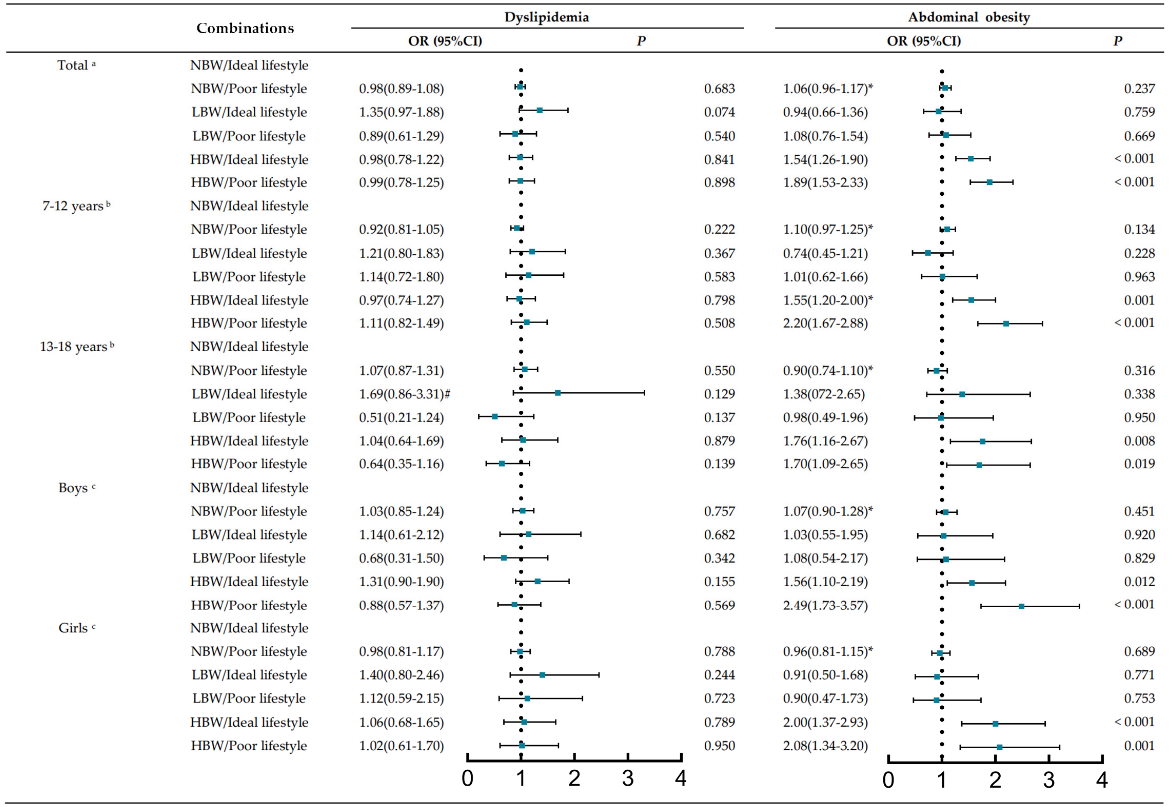

	Combinations	Dyslipidemia OR (95%CI)	Dyslipidemia *P*	Abdominal obesity OR (95%CI)	Abdominal obesity *P*
Total [a]	NBW/Ideal lifestyle				
	NBW/Poor lifestyle	0.98(0.89-1.08)	0.683	1.06(0.96-1.17)*	0.237
	LBW/Ideal lifestyle	1.35(0.97-1.88)	0.074	0.94(0.66-1.36)	0.759
	LBW/Poor lifestyle	0.89(0.61-1.29)	0.540	1.08(0.76-1.54)	0.669
	HBW/Ideal lifestyle	0.98(0.78-1.22)	0.841	1.54(1.26-1.90)	< 0.001
	HBW/Poor lifestyle	0.99(0.78-1.25)	0.898	1.89(1.53-2.33)	< 0.001
7-12 years [b]	NBW/Ideal lifestyle				
	NBW/Poor lifestyle	0.92(0.81-1.05)	0.222	1.10(0.97-1.25)*	0.134
	LBW/Ideal lifestyle	1.21(0.80-1.83)	0.367	0.74(0.45-1.21)	0.228
	LBW/Poor lifestyle	1.14(0.72-1.80)	0.583	1.01(0.62-1.66)	0.963
	HBW/Ideal lifestyle	0.97(0.74-1.27)	0.798	1.55(1.20-2.00)*	0.001
	HBW/Poor lifestyle	1.11(0.82-1.49)	0.508	2.20(1.67-2.88)	< 0.001
13-18 years [b]	NBW/Ideal lifestyle				
	NBW/Poor lifestyle	1.07(0.87-1.31)	0.550	0.90(0.74-1.10)*	0.316
	LBW/Ideal lifestyle	1.69(0.86-3.31)#	0.129	1.38(072-2.65)	0.338
	LBW/Poor lifestyle	0.51(0.21-1.24)	0.137	0.98(0.49-1.96)	0.950
	HBW/Ideal lifestyle	1.04(0.64-1.69)	0.879	1.76(1.16-2.67)	0.008
	HBW/Poor lifestyle	0.64(0.35-1.16)	0.139	1.70(1.09-2.65)	0.019
Boys [c]	NBW/Ideal lifestyle				
	NBW/Poor lifestyle	1.03(0.85-1.24)	0.757	1.07(0.90-1.28)*	0.451
	LBW/Ideal lifestyle	1.14(0.61-2.12)	0.682	1.03(0.55-1.95)	0.920
	LBW/Poor lifestyle	0.68(0.31-1.50)	0.342	1.08(0.54-2.17)	0.829
	HBW/Ideal lifestyle	1.31(0.90-1.90)	0.155	1.56(1.10-2.19)	0.012
	HBW/Poor lifestyle	0.88(0.57-1.37)	0.569	2.49(1.73-3.57)	< 0.001
Girls [c]	NBW/Ideal lifestyle				
	NBW/Poor lifestyle	0.98(0.81-1.17)	0.788	0.96(0.81-1.15)*	0.689
	LBW/Ideal lifestyle	1.40(0.80-2.46)	0.244	0.91(0.50-1.68)	0.771
	LBW/Poor lifestyle	1.12(0.59-2.15)	0.723	0.90(0.47-1.73)	0.753
	HBW/Ideal lifestyle	1.06(0.68-1.65)	0.789	2.00(1.37-2.93)	< 0.001
	HBW/Poor lifestyle	1.02(0.61-1.70)	0.950	2.08(1.34-3.20)	0.001

Figure S8. Associations between dyslipidemia, abdominal obesity and combinations of birth weight and lifestyle stratified by age and sex groups

Note: Model a was adjusted for age, sex, residence, delivery mode, single-child status, parental smoking, parental education and school effect. Model b was adjusted for sex, residence, delivery mode, single-child status, parental smoking, parental education and school effect. Model c was adjusted for age, residence, delivery mode, single-child status, parental smoking, parental education and school effect. NBW, normal birth weight; LBW, low birth weight; HBW, high birth weight; CMRFs, cardio-metabolic risk factors; OR, odds ratio; CI, confidence interval. #, the odds ratio was differed from the group of LBW/Poor lifestyle statistically significant. *, the odds ratio was differed from the group of HBW/Poor lifestyle statistically significant.

	Combinations	Clustered CMRFs	
		OR (95%CI)	*P*
Total [a]	NBW/Ideal lifestyle		
	NBW/Poor lifestyle	1.04(0.83-1.29)	0.754
	LBW/Ideal lifestyle	2.00(1.07-3.72)	0.029
	LBW/Poor lifestyle	0.95(0.41-2.19)	0.896
	HBW/Ideal lifestyle	0.76(0.43-1.33)	0.327
	HBW/Poor lifestyle	1.74(1.13-2.68)	0.012
7-12 years [b]	NBW/Ideal lifestyle		
	NBW/Poor lifestyle	0.89(0.66-1.21)	0.470
	LBW/Ideal lifestyle	1.02(0.36-2.84)	0.977
	LBW/Poor lifestyle	1.38(0.54-3.53)	0.506
	HBW/Ideal lifestyle	1.07(0.56-2.04)	0.838
	HBW/Poor lifestyle	2.37(1.39-4.05)	0.002
13-18 years [b]	NBW/Ideal lifestyle		
	NBW/Poor lifestyle	1.18(0.77-1.81)	0.442
	LBW/Ideal lifestyle	3.57(1.23-10.37)	0.019
	LBW/Poor lifestyle	1.00(0.62-1.48)	0.970
	HBW/Ideal lifestyle	0.36(0.08-1.58)	0.177
	HBW/Poor lifestyle	1.06(0.40-2.83)	0.907
Boys [c]	NBW/Ideal lifestyle		
	NBW/Poor lifestyle	1.33(0.91-1.95)	0.135
	LBW/Ideal lifestyle	2.78(1.02-7.62)	0.047
	LBW/Poor lifestyle	0.56(0.08-4.23)	0.576
	HBW/Ideal lifestyle	0.76(0.29-1.98)	0.574
	HBW/Poor lifestyle	1.19(0.51-2.74)	0.689
Girls [c]	NBW/Ideal lifestyle		
	NBW/Poor lifestyle	0.68(0.41-1.13)*	0.135
	LBW/Ideal lifestyle	1.60(0.46-5.55)	0.462
	LBW/Poor lifestyle	1.19(0.26-5.41)	0.818
	HBW/Ideal lifestyle	1.38(0.52-3.68)	0.520
	HBW/Poor lifestyle	1.92(0.72-5.10)	0.191

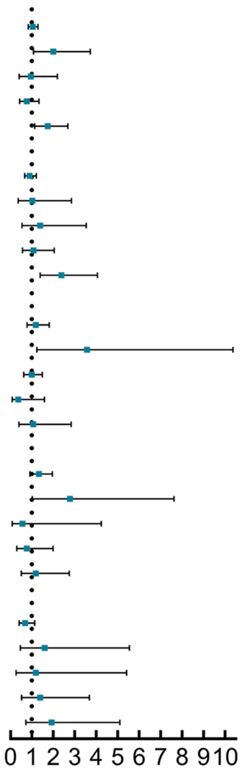

Figure S9. Associations between clustered CMRFs and combinations of birth weight and lifestyle stratified by age and sex groups

Note: Model a was adjusted for age, sex, residence, delivery mode, single-child status, parental smoking, parental education and school effect. Model b was adjusted for sex, residence, delivery mode, single-child status, parental smoking, parental education and school effect. Model c was adjusted for age, residence, delivery mode, single-child status, parental smoking, parental education and school effect. NBW, normal birth weight; LBW, low birth weight; HBW, high birth weight; CMRFs, cardio-metabolic risk factors; OR, odds ratio; CI, confidence interval. #, the odds ratio was differed from the group of LBW/Poor lifestyle statistically significant. *, the odds ratio was differed from the group of HBW/Poor lifestyle statistically significant.
